# Prognostic value of ABO blood group in a Chinese population in Northwest China region with curatively resected rectal cancer

**DOI:** 10.7150/jca.32407

**Published:** 2019-10-21

**Authors:** Jiangpeng Wei, Ying Zhang, Jianyong Zheng, Xiangying Feng, Xiaomin Wang, Kunli Du, Weizhong Wang, Guosheng Wu, Qingchuan Zhao, Daiming Fan, Xiaohua Li

**Affiliations:** 1 State Key Laboratory of Cancer Biology, National Clinical Research Center for Digestive Diseases and Xijing Hospital of Digestive Diseases, Fourth Military Medical University, Xi'an, Shaanxi, China.; 2 Department of radiotherapy, Xijing Hospital, Fourth Military Medical University, Xi'an, Shaanxi, China.

**Keywords:** ABO blood group, rectal cancer, retrospective cohort, survival

## Abstract

A positive association between the ABO blood types and survival has been suggested in several malignancies. However, little is known about the relationship between ABO blood group and survival in rectal cancer patients. The aim of this study was to assess the role of the ABO blood types in predicting the prognosis of a Chinese population in Northwest China region with curatively resected rectal cancer. We retrospectively analyzed 1613 consecutive patients who underwent curative surgery for rectal cancer between June, 2011 and December, 2016. The relationship between the ABO blood types and overall survival (OS) was analyzed. The median follow-up period of the 1613 rectal cancer patients was 69.6 months with 1427 alive. There was a significance difference of survival among ABO blood groups (P=0.007). The mean overall survival (OS) of the blood type B patients was 70.8 months, O was 64.3, whereas the mean OS of the AB and A blood type patients was significantly lower, 58.4 months and 59.7 months respectively (P=0.007, log-rank test). Compared with patients with A and AB blood types, patients with blood type B and O were more likely to have better survival(P=0.001). A blood groups were associated with significantly decreased overall survival in rectal cancer patients (hazard ratio = 1.263; 95% confidence interval = 0.776-2.054, P =0.010). In order to confirm our above results, we performed the same investigation in an independent cohort from another hospital of 505 Chinese patients and get the similar results. Our study showed that ABO blood group is associated with survival in Northwest Chinese patients with rectal cancer and the blood type B and O were favourable prognostic factors for patients with rectal cancer.

## Introduction

Colorectal cancer is the third-most commonly diagnosed cancer in males and the second-most common in female 1. Each year, more than 1.2 million new cases of colorectal cancer are diagnosed worldwide 2. Previously, colorectal cancer has its highest incidence in Western Europe and North America, but recently, the mortality and morbidity of colorectal cancer rapidly grows in the Chinese population in Northwest China region. According to the latest Cancer Statistics of China, colorectal cancer is the fifth newly diagnosed cancer among men, and the fourth among women in China 3. Among those colorectal cancer patients, rectal cancer represents 40 percent of colorectal cancers. While curative surgery is the only option for long term survival, rectal cancer spreads more frequently to the thoracic organs, bone and nervous system and approximately 20% of rectal cancer patients lose opportunity for radical surgery on account of metastases 4, 5.

Although prognostic factors for rectal cancer have conducted intensive studies, including in the field of molecular biology, but for the prognosis of rectal cancer can be different despite similar stages and grades 6. A better understanding of an ideal biomarker with readily available, inexpensive and reproducible of rectal cancer could improve the prognosis of patients and provide appropriate therapy strategies. Recently, the correlation between the ABO blood type and other malignancies, such as breast cancer, pancreatic cancer, lung cancer, esophageal squamous cell carcinoma, colon cancer, nasopharyngeal carcinoma, and obstetric cancers, has been continuously reported 7-14.

Previously Hamed et al. 15 enrolled 1025 colorectal cancer patients in two large prospective cohorts and observed the relationship between ABO blood group and risk of colorectal cancer, their results showed that the ABO blood group didn't have any association with risk of colorectal cancer. Well-known, many genetic and environmental risk factors were defined for cancer, including smoking, obesity, a higher-fat diet, rectal polyp and a family history. However, up to now, studies of the impact of ABO blood group on the survival of the Chinese population in Northwest China region with rectal cancer remained uncertain. Therefore, the aim of this retrospective analysis was to analyze the relationship between ABO blood type and the survival of rectal cancer patients in a Chinese population in Northwest China region as there is an interpopulation variation for this condition.

## Material and Methods

### Patient selection

The retrospective study included 1613 patients who were diagnosed with rectal adenocarcinoma and treated surgically between June, 2011 and December, 2016 at Division of Gastrointestinal Surgery, First Affiliated Hospital of Air Force Military Medical University. Enrolled patients were histologically confirmed and without distant metastasis. Patients with one of the following features, (stage IV) rectal cancer, with more than one primary cancer, with R1 or R2 resection, or death from postoperative complications, were excluded from our study. Other patients with missing data were also excluded. Patients were considered eligible only when the following data were available. Tumor differentiation grades were defined according to the World Health Organization criteria. Cancer staging was based on the American Joint Committee on Cancer Staging system (AJCC, 2002; Greene, American Joint Committee on Cancer, American Cancer Society, 2002). The study was approved by the ethics committee of First Affiliated Hospital of Air Force Military Medical University. All patients provided written consent for storage of their information in the hospital database, and for the research use of the information.

### Follow-up and outcome

Each patient was followed up periodically until death or April 2017(every 3 mouth for the first 2 y, and every 6 mouth up to the fifth year) after surgery. The follow-up cycles varied from 3-6mo, with a median of 69.6 months. The follow-up visits consisted of a physical examination and laboratory studies at least every 6 months or when clinically indicated. The endpoint of the study was overall survival (OS). OS was calculated as the period from the date of diagnosis to the date of death from any cause or the date of last follow-up. Survival status was verified again using the best available methods, including checking clinical attendance records and direct telecommunication with the patients or their families.

### A validation cohort

An independent cohort included 505 rectal adenocarcinoma patients who were diagnosed and treated surgically between December, 2012 and December, 2015 at Division of Gastrointestinal Surgery, Second Affiliated Hospital of Air Force Military Medical University, were used for confirm the above results. The admission and exclusion condition of the enrolled patients were the same as before. All patients provided written consent for storage of their information in the hospital database, and for the research use of the information. Each patient was followed up periodically until death or April 2017 (every 3 mouth for the first 2 y, and every 6 mouth up to the fifth year) after surgery. The follow-up cycles varied from 3-6mo, with a median of 59.6 months. The follow-up visits consisted of a physical examination and laboratory studies at least every 6 months or when clinically indicated. The endpoint of the study was overall survival (OS). OS was calculated as the period from the date of diagnosis to the date of death from any cause or the date of last follow-up. Survival status was verified again using the best available methods, including checking clinical attendance records and direct telecommunication with the patients or their families.

### Statistical analysis

Chi-square test was used to compare categorical variables. ANOVA test was used to compare continuous variable, if they have no homogeneity of variance, then Kruskal Wallis Test will be used. The survival rates were evaluated by Kaplane-Meier survival analysis, and their significance was calculated by the log-rank test. Univariate and multivariate Cox regression analyses were performed. Multivariable analyses were performed for factors which were significantly associated with OS in univariate analyses. All the statistical analyses were conducted using IBM SPSS 20.0 software. A P value <0.05 was considered to be statistically significant.

## Results

### ABO blood group and clinicopathologic characteristics of the rectal cancer patients

A total of 1613 eligible patients with rectal cancer were analyzed at the gastrointestinal surgery in First Affiliated Hospital of Air Force Military Medical University from June, 2011 to December, 2016. Clinicopathologic characteristics of all subjects stratified by ABO blood group were displayed in Table 1. Based on the seventh edition of the TNM-UICC/AJCC classification system, the numbers of patients with stage I, II, and III disease were 439 (27.2%), 427 (26.5%), and 747(46.3%), respectively. The median age of the patients was 59 years (range, 19-86 years). Among the 1613 subjects, 411 (25.5%) were blood group A, 502 (31.1%) were blood group B, 545(33.8%) were blood group O, and the remaining 155 (9.6%) were blood group AB. No significant difference was found regarding gender, age, tumor size, tumor location, BMI, degree of differentiation, lymphatic/vascular invasion, perineural invasion, N stage, smoking history, drinking history, CEA level, CA199 level and CA125 level.

### Association between the clinical prognosis of rectal cancer patients and ABO blood groups

**Figure 1:**
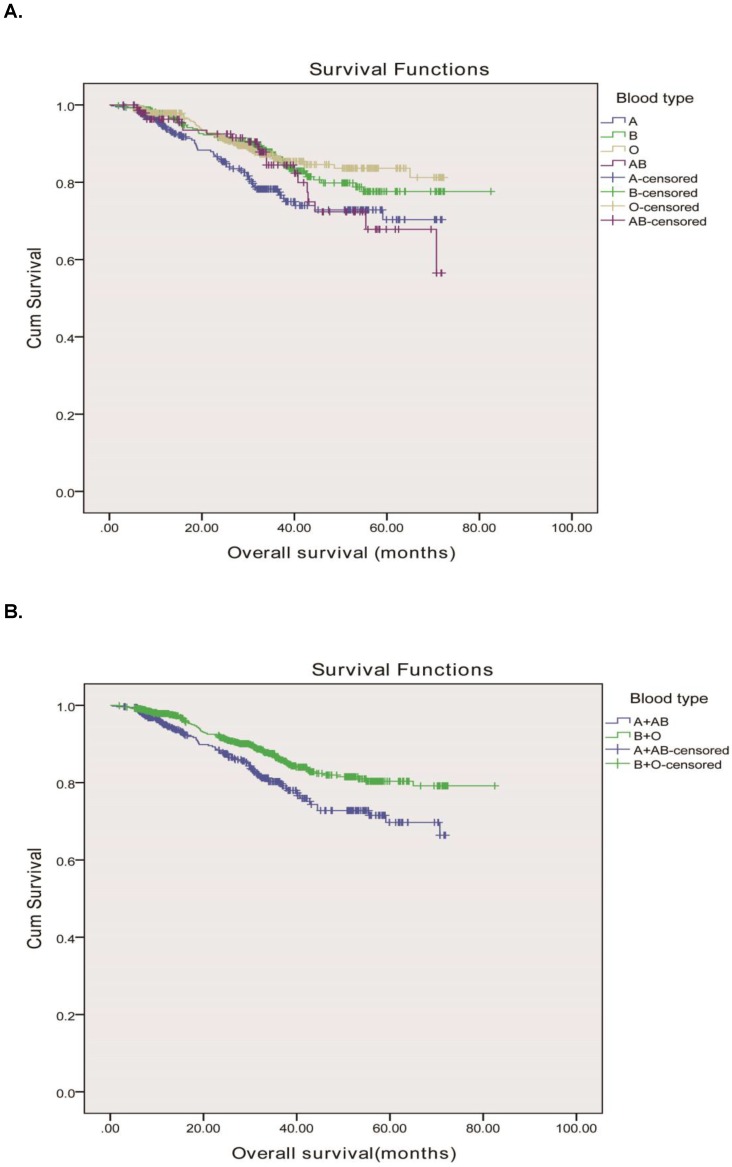
Overall survival, by ABO blood type, among patients in our study. A, Survival curve of the 1613 rectal adenocarcinoma patients according to the ABO blood groups. B, Survival curve of patients with blood types B+O and A + AB. We divided the whole group of patients into two subgroups, patients with B and O blood types and patients with A and AB blood groups. The two survival curve separated, and had significant difference. The 5-year overall survival was 90.3 vs. 85.2%, respectively, P = 0.001.

The median follow-up period of the 1613 rectal cancer patients was 69.6 months with 1427 (88.5%) alive and 186 (11.5%) dead from cancer-related diseases at the final clinical follow-up. The 5-year overall survival rates for rectal adenocarcinoma patients with the A, B, O and AB blood types were 84.9%, 89.2%, 91.2% and 85.8%, respectively. The ABO blood groups were closely associated with OS according to the Kaplane-Meier analysis (P=0.007) Figure 1A. OS was longer in rectal cancer patients with the group B (median, 70.8mo, 95% CI, 67.9-73.6 months) than in those with the group A (median, 58.4mo, 95% CI, 55.4-61.3 months), group O (median, 64.3mo, 95% CI, 62.2-66.5months), and group AB (median, 59.7mo, 95% CI, 55.2-64.2 months) (P = 0.007). Meanwhile, we found that the blood types B and O seemed to have a higher survival than blood types A and AB, therefore, we divided the whole group of patients into two subgroups. The subgroup analysis indicated that patients of blood group B and O had a longer OS compared to those of blood group A and AB (71.6 months vs. 58.8 months, P = 0.001) as shown in Figure 1B.

Then the relationship between ABO blood type and survival based on patients' clinicopathologic characteristics were examined. These analysis showed that ABO blood group could distinguish OS when stratified by gender (Female, P=0.014), age(<60, P=0.028), smoking history (no smoking, P=0.017) ,drinking history (no drinking, P=0.046), CEA (normal, P=0.007), CA199 (elevated, P=0.013), CA-125 (normal, P=0.004), tumor location (tumor height <6 cm, P=0.006),tumor size (≥3.4cm, P=0.009), pathology (well differentiated, P<0.001; poorly differentiated, P=0.014), perineural invasion (yes, P=0.023), vascular invasion (no, P=0.004), pT status (T1, P=0.026; T3, P=0.003), pN status (no, P=0.001).

To determine whether ABO blood type could serve as an independent prognostic factor, we examined OS using the Cox proportional hazards model. Univariate analysis was used to evaluated the influence of the patients' gender, age, body mass index(BMI),tumor location, pathology differentiation, lymphatic/vascular invasion, perineural invasion, T status, N status, TNM stage, smoking history, drinking history, ABO blood type, tumor size, serum CEA , CA199 and CA125 level on OS (Table 2). Our results showed that the poorly differentiated histology (P<0.001), lower BMI (P=0.007), lymphatic/vascular invasion (P<0.001), advanced pT stages (P <0.001), advanced pN stages (P<0.001), advanced tumor stages (P <0.001), ABO blood type (P=0.010) ,lower tumor height (P<0.001), bigger tumor size (P<0.001), serum CEA elevation (P = 0.011) , serum CA-199 elevation (P<0.001) and serum CA-125 elevation (P<0.001) were all significantly associated with shorter survival. Furthermore, the significant parameters in univariate analysis were also performed by multivariate analyses. The results revealed that lower BMI (P=0.031), lower tumor height (P=0.021), poorly differentiated histology (P=0.005), lymphatic/vascular invasion (P=0.017), advanced pN stages (P=0.029), ABO blood type (P=0.010), serum CA-199 elevation (P=0.022) and serum CA-125 elevation (P<0.001) were independent, significant predictors for OS (Table 2).

### Validation in an independent cohort

**Figure 2:**
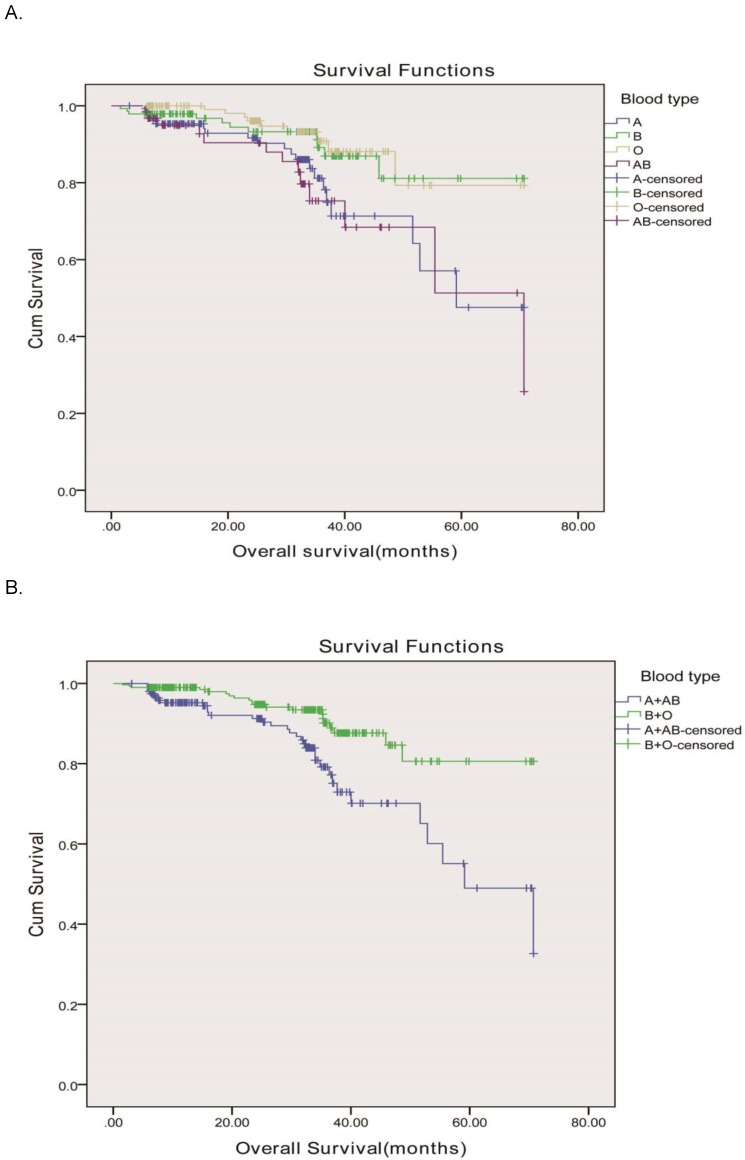
Overall survival, by ABO blood type, among patients in our study. A, Survival curve of the 505 rectal adenocarcinoma patients according to the ABO blood groups. (P = 0.003)B, Survival curve of patients with blood types B+O and A + AB. We divided the whole group of patients into two subgroups, patients with B and O blood types and patients with A and AB blood groups. The two survival curve separated, and had significant difference. The 5-year overall survival was 86.8 vs. 80.6%, respectively, P <0.001.

In order to validate our results, we used an independent cohort from another hospital for further confirm and the clinicopathologic characteristics of all subjects stratified by ABO blood group were displayed in Table 3. There were 505 enrolled rectal cancer patients, 287 (56.8%) male and 218 (43.2%) female, aged from 23-88 y (the median age was 69 y). Based on the seventh edition of the TNM-UICC/AJCC classification system, the numbers of patients with stage I, II, and III disease were 141 (27.9%), 124 (24.6%), and 240 (47.5%), respectively. The ABO blood groups were distributed as 140 patients with the blood group A, 142 patients with the blood group B, and 161 patients with the blood group O, and 62 patients with the blood group AB. Our results showed that the ABO blood groups were closely associated with OS according to the Kaplane-Meier analysis (P=0.003) Figure 2A. OS was longer in rectal cancer patients with the group O (median, 63.8mo, 95% CI, 59.1-68.7 months) than in those with the group B (median, 63.3mo, 95% CI, 58.9-67.7 months), group A (median, 54.7mo, 95% CI, 48.9-60.5months), and group AB (median, 54.3mo, 95% CI, 46.3-62.3 months) as shown in Figure 2A. Blood types B and O in this independent cohort also showed a higher survival than blood types A and AB. Therefore, we divided the whole group of patients into two subgroups. The subgroup analysis indicated that patients of blood group B and O also had a longer OS compared to those of blood group A and AB (63.7 months vs. 54.5months, P < 0.001) as shown in Figure 2B, similar to the results showed in our hospital.

Furthermore, this independent cohort analysis date also showed that the poorly differentiated histology (P=0.005), lymphatic/vascular invasion (P=0.003), advanced pT stages (P =0.042), advanced pN stages (P=0.006), advanced tumor stages (P =0.003), ABO blood type (P=0.006) ,lower tumor height (P=0.012), bigger tumor size (P=0.019), serum CA-199 elevation (P=0.038) and serum CA-125 elevation (P=0.023) were all significantly associated with shorter survival. Meanwhile, the significant parameters in univariate analysis were further analyzed in multivariate analyses. Our results showed that the ABO blood groups were independent predictors for OS (group A: hazard ratio [HR] = 0.832, 95% confidence interval [CI] = 0.416-1.666; group B: hazard ratio [HR] = 0.368, 95% confidence interval [CI] = 0.165-0.823; group O: hazard ratio [HR] = 0.299, 95% confidence interval [CI] = 0.128-0.699; group AB: hazard ratio [HR] = 1, 95% confidence interval [CI] = Reference). Our results also revealed that lower tumor height (P=0.004), poorly differentiated histology (P=0.002), lymphatic/vascular invasion (P=0.021), advanced pT stages (P =0.012), advanced pN stages (P=0.023), advanced tumor stages (P =0.015), ABO blood type (P=0.008) and serum CA-125 elevation (P=0.031) were independent, significant predictors for OS (Table 4). From this independent cohort, we got the similar conclusion, which further confirmed the ABO blood group is associated with survival in Chinese patients with rectal cancer and the blood type B and O were favourable prognostic factors for patients with rectal cancer.

## Discussion

In the present study, we investigated the value of ABO blood group in predicting the prognosis of patients who underwent radical surgery for rectal cancer. We observed significantly better survival for participants with blood group B and O compared with A and AB blood group participants. Using multivariate analysis, significant associations with rectal cancer survival were found BMI, tumor location, poorly differentiated histology, lymphatic/vascular invasion, advanced pN stages, CA125, CA19-9. Our study is the first study that investigated this relationship in a Chinese population in Northwest China region and we found that blood type is an independent factor affecting the prognosis of patients with rectal cancer.

The ABO blood group is by far the most important among human blood group systems 16. The ABO blood group system is based on expression of two antigens, A and/or B on the surface of the red blood cell; because expression of these antigens is codominant, patients may have type A, type B or type AB expression patterns. Lack of expression of either antigen results in the O phenotype 17. For several decades, a role for ABO blood group antigens in the development of cancer has been suspected, but the results have been inconsistent. In a study conducted by Nozoe and Colleagues in 284 patients, non-O blood groups correlated with poorly differentiated grades of the tumour and AB blood group was associated with advanced stage and large tumour size, the significance of ABO blood group distribution might be associated with biological behavior of gastric adenocarcinoma patients, but no correlation was found between different ABO blood groups and overall survival 18. By contrast, in another study the B/O group was found to independently correlate with unfavourable survival among patients who had ever smoked 11. A large, prospective, population-based study has consistently documented an increased risk of gastric cancer in individuals with blood type A 19. However, it was found not to be a prognosis factor for patients with gastric adenocarcinoma. In a recent study conducted in 1555 patients with surgically resected colon cancer, AB blood type were more likely to have a better survival than patients with non-AB blood types^12^, but adverse consequences were found in our study. This might be caused by our small number of subjects as well as by interpopulation variation. The exact biological theories elaborating the link between ABO blood group and cancer are still insufficient. Researchers involved in blood group ABO antigens and their genes, developed by Hakomori have shown that human blood group antigens are expressed on the surface of red blood cells and other tissues, including cells of the gastrointestinal tract^20^, broncho pulmonary, skin, urogenital epithelial and kidney^21^. These glycoconjugates may participate in modifying intercellular adhesion, membrane signaling, and immune surveillance, which could in turn influence tumorigenesis 20.

Although the mechanism for an association between blood group and rectal cancer risk is unknown, several plausible hypotheses exist. The normal ABO antigen is lost in cancer patients, and new tumor antigens are acquired. In colon carcinomas, 50% of tumors of the proximal colon demonstrated loss of antigen expression, although expression was retained in the adjacent tissues. Of note, in tumors of the distal colon, antigens, while undetectable in the adjacent tissues, were expressed in tumor cells 22, 23, thus, a structural change in the ABO antigen may occurs in rectal cancer. A likely mechanism for the alteration of ABH antigen expression in colorectal tissue is the loss or reduction of the glycosyltransferase enzymes required for the synthesis of ABH determinants 24. Modified expression of blood group antigens on cancer cells may influence tumorigenesis by altering glycosyltransferase specificity 25 or increasing cell motility, resistance to apoptosis and immune escape 21. It is therefore possible that a specific blood type may enhance disease progression or survival 21, 26. A number of single nucleotide polymorphism studies have suggested that ABO gene may influence the systemic chronic inflammatory response that is closely related to carcinogenesis by regulating the serum levels of several circulating adhesion molecules and plasma inflammatory markers 27, such as tumor necrosis factor alpha 28, 29, soluble intercellular adhesion molecule (ICAM)-1 30, 31, E-selectin 32, 33, and P-selectin 30. All these adhesion molecules are important mediators of chronic inflammation and immune cell recruitment. They may, therefore, provide a biological basis for the postulated influence of ABO on cancer survival, by directly linking ABO blood group and tumour initiation and spread 34.

We acknowledge the limitations of our retrospective analysis. The retrospective nature of our study greatly increased the possibility that factors related to blood group influenced which cases were included in the analysis. Additional shortcoming of our analysis include the insufficient study population, the short follow-up percentage and the unavailability of detailed covariate data, which cannot allowed us to examine confounding and effect modification by several exposures of interest. Our study population was composed of all Chinese population in Northwest China region, which somewhat limits the generalizability of our results. Accordingly, the results of further investigations including more diverse populations (white/black/brown participants) from other institutes are needed to confirm our findings. In addition, even all patients had computed tomography scans of the chest and abdomen at the time of diagnosis, but not for brain, and it is possible that some patients had asymptomatic disease at the time of primary treatment. However, this would have had a negative influence on survival. Furthermore, hospital-based control populations may not be representative of the blood group distribution in the general population if the conditions leading to hospitalization are associated with blood group.

## Figures and Tables

**Table 1:** Characteristics according to ABO blood type

Patient characteristics	Total	ABO				P-values
		A	B	O	AB	*P*^a^
**Total**	**1613**	**411**	**502**	**545**	**155**	
**Gender**						0.495
Male	884	237	265	295	87	
Female	729	174	237	250	68	
**Age ^b^(years)**						0.466
<60	807	225	250	266	66	
≥60	806	186	252	279	89	
**BMI^ b^, kgm^2^**						0.231
<18.5	134	39	41	46	8	
18.5-23.9	878	227	284	291	76	
≥24	601	145	177	208	71	
**Location^c^**						0.970
tumour height >12 cm	91	22	29	30	10	
tumour height 6-12 cm	667	174	201	224	68	
tumour height <6 cm	855	215	272	291	77	
**Pathology(adenocarcinoma)**						0.539
Well differentiated	220	59	64	78	19	
Moderately differentiated	1200	292	383	406	119	
Poorly differentiated	193	60	55	61	17	
**Lymphatic/vascular invasion**						0.785
Yes	547	139	168	192	48	
No	1066	272	334	353	107	
**PNI**						0.836
Yes	1013	261	307	347	98	
No	600	150	195	198	57	
**pT status**						0.027
pT1	165	36	42	65	22	
pT2	405	92	120	160	33	
pT3	962	260	316	292	94	
pT4	81	23	23	28	6	
**pN status**						0.057
pN0	829	198	259	295	77	
pN1	517	141	171	148	57	
pN2	267	72	72	102	21	
**pTNM stage**						0.043
Stage I	439	102	122	174	41	
Stage II	427	104	152	130	41	
Stage III	747	205	228	241	73	
**Smoking history**						0.618
Yes	461	112	138	167	44	
No	1152	299	364	378	111	
**Drinking history**						0.222
Yes	331	80	91	125	35	
No	1282	331	411	420	120	
**Tumor size**		A	B	O	AB	0.253
≥3.4		272	315	326	96	
<3.4		139	187	219	59	
**CEA**						0.875
Normal	1098	281	347	364	106	
Elevated	515	130	155	181	49	
**CA199**						0.231
Normal	1461	363	457	496	145	
Elevated	152	48	45	49	10	
**CA125**						0.880
Normal	1561	396	485	530	150	
Elevated	52	15	17	15	5	

Abbreviations: BMI=body mass index; pN status=pathological node status; pT statue=pathological tumour status; pTNM status=pathological tumour-node-metastasis stage. a: χ^2^ test (A blood type vs B blood type vs O blood type vs AB blood type). b: Chinese definition. c: NCOTARGET. 2015;6(34):36884-9.

**Table 2:** Univariate and multivariate Cox regression analysis for overall survival in patients with rectal cancer

Characteristics	Univariate analysis			Multivariate analysis		
	HR	95% CI	*P*^a^	HR	95% CI	*P*^a^
**Gender**			0.188			—
Male	1.00	Reference		—	—	
Female	0.819	0.609-1.102		—	—	
**Age^b^ (years)**			0.472			—
<60	1.111	0.833-1.482		—	—	
≥60	1	Reference		—	—	
**BMI^b^, kgm^2^**			0.007			0.031
<18.5	2.168	1.369-3.432		1.808	1.127-2.899	
18.5-23.9	1.197	0.864-1.657		1.045	0.751-1.454	
≥24	1	Reference		1	Reference	
**Location^c^**			0.050			0.021
tumor height >12 cm	0.888	0.465-1.695		0.826	0.429-1.594	
tumor height 6-12 cm	0.685	0.504-0.932		0.639	0.465-0.877	
tumor height <6 cm	1	Reference		1	Reference	
**Pathology (adenocarcinoma)**			<0.001			0.005
Well differentiated	0.265	0.161-0.437		0.544	0.315-0.940	
Moderately differentiated	0.346	0.243-0.492		0.537	0.369-0.782	
Poorly differentiated	1	Reference		1	Reference	
**lymphatic/vascular invasion**			<0.001			0.017
Yes	1	Reference		1	Reference	
No	0.399	0.299-0.534		0.656	0.464-0.927	
**PNI**			0.387			—
Yes	1	Reference		—	—	
No	0.879	0.655-1.179		—	—	
**pT status**			<0.001			0.092
pT1	0.196	0.085-0.452		0.380	0.25-1.159	
pT2	0.252	0.137-0.465		0.394	0.174-0.893	
pT3	0.662	0.405-1.083		0.927	0.551-1.561	
pT4	1	Reference		1	Reference	
**pN status**			<0.001			0.029
pN0	1.00	Reference		1.00	Reference	
pN1	2.093	1.468-2.985		0.369	0.125-1.087	
pN2	4.502	3.138-6.458		0.628	0.432-0.914	
**pTNM stage**			<0.001			0.651
Stage I	0.253	0.162-0.395		1.624	0.463-5.703	
Stage II	0.441	0.306-.0637		1.112	0.385-3.216	
Stage III	1.00	Reference		1.00	Reference	
**Smoking history**			0.930			—
Yes	1.014	0.739-1.392		—	—	—
No	1.00	Reference		—	—	
**Drinking history**			0.479			—
Yes	1.134	0.804-1.600		—	—	
No	1.00	Reference		—	—	
**ABO blood type**			0.010			0.013
A	1.263	0.776-2.054		1.186	0.720-1.954	
B	0.773	0.471-1.269		0.709	0.429-1.172	
O	0.696	0.420-1.152		0.689	0.412-1.152	
AB	1	Reference		1	Reference	
**Tumor size(cm)**			<0.001	—	—	0.060
<3.4	0.525	0.377-0.731		0.714	0.503-1.014	
≥3.4	1	Reference		1	Reference	
**Serum CEA**			0.011			0.509
Normal	0.680	0.508-0.912		0.902	0.902-0.663	
Elevated	1	Reference		1	Reference	
**CA19-9**			<0.001			0.022
Normal	0.408	0.283-0.590		0.631	0.426-0.934	
Elevated	1	Reference		1	Reference	
**CA125**			<0.001			<0.001
Normal	0.299	0.184-0.486		0.287	0.174-0.474	
Elevated	1	Reference		1	Reference	

Abbreviations: BMI=body mass index; 95% CI=95% confidence interval; HR=hazard ratio; pTNM stage=pathological tumour-node-metastasis stage. a: Cox proportional hazards model. b: Chinese definition. c: NCOTARGET. 2015;6(34):36884-9.

**Table 3:** Characteristics according to ABO blood type

Patient characteristics	Total	ABO				P-values
		A	B	O	AB	*P*^a^
**Total**	**505**	**140**	**142**	**161**	**62**	
**Gender**						0.770
Male	287	82	83	86	36	
Female	218	58	59	75	26	
**Age ^b^(years)**						0.054
<60	233	71	59	82	21	
≥60	272	69	83	79	41	
**BMI^ b^, kgm^2^**						0.721
<18.5	36	9	13	10	4	
18.5-23.9	282	83	81	87	31	
≥24	187	48	48	64	27	
**Location^c^**						0.339
tumour height >12 cm	30	4	7	15	4	
tumour height 6-12 cm	209	62	62	60	25	
tumour height <6 cm	266	74	73	86	33	
**Pathology(adenocarcinoma)**						0.526
Well differentiated	72	18	17	28	9	
Moderately differentiated	376	105	113	115	43	
Poorly differentiated	57	17	12	18	10	
**Lymphatic/vascular invasion**						0.868
Yes	323	88	90	107	38	
No	182	52	52	54	24	
**PNI**						0.723
Yes	169	51	49	49	20	
No	336	89	93	112	42	
**pT status**						0.024
pT1	60	15	12	25	8	
pT2	119	29	34	50	6	
pT3	289	85	86	74	44	
pT4	37	11	10	12	4	
**pN status**						0.277
pN0	247	61	70	91	25	
pN1	171	54	47	45	25	
pN2	87	25	25	25	12	
**pTNM stage**						0.038
Stage I	141	36	37	58	10	
Stage II	124	29	41	37	17	
Stage III	240	75	64	66	35	
**Smoking history**						0.390
Yes	359	101	107	107	44	
No	146	39	35	54	18	
**Drinking history**						0.281
Yes	400	114	118	122	46	
No	105	26	24	39	16	
**Tumor size**						0.155
≥3.4	194	49	48	73	24	
<3.4	311	91	94	88	38	
**CEA**						0.117
Normal	333	90	105	99	39	
Elevated	172	50	37	62	23	
**CA199**						0.889
Normal	469	129	132	149	59	
Elevated	36	11	10	12	3	
**CA125**						0.858
Normal	488	134	137	157	60	
Elevated	17	6	5	4	2	

Abbreviations: BMI=body mass index; pN status=pathological node status; pT statue=pathological tumour status; pTNM status=pathological tumour-node-metastasis stage. a:χ2 test (A blood type vs B blood type vs O blood type vs AB blood type). b: Chinese definition. c: NCOTARGET. 2015;6(34):36884-9.

**Table 4:** Univariate and multivariate Cox regression analysis for overall survival in patients with colon cancer

Characteristics	Univariate analysis			Multivariate analysis		
	HR	95% CI	*P*^a^	HR	95% CI	*P*^a^
**Gender**			0.522			—
Male	1.00	Reference		—	—	
Female	0.29	0.468-1.470		—	—	
**Age^b^ (years)**			0.929			—
<60	1	Reference		—	—	
≥60	0.929	0.600-1.751		—	—	
**BMI^b^, kgm^2^**			0.971			—
<18.5	1	Reference		—	—	
18.5-23.9	0.916	0.312-2.690		—	—	
≥24	0.937	0.531-1.653		—	—	
**Location^c^**			0.012			0.004
tumor height >12 cm	1	Reference		1	Reference	
tumor height 6-12 cm	1.081	0.209-3.721		1.080	0.595-1.961	
tumor height <6 cm	1.176	0.6812.031		1.126	0.236-5.365	
**Pathology (adenocarcinoma)**			0.005			0.002
Well differentiated	1	Reference		1	Reference	
Moderately differentiated	1.144	0.295-4.436		2.338	0.539-10.135	
Poorly differentiated	1.879	0.583-6.054		3.647	0.332-1.260	
**lymphatic/vascular invasion**			0.003			0.021
Yes	1	Reference		1	Reference	
No	0.441	0.257-0.758		0.647	0.3324-1.260	
**PNI**			0.522			—
Yes	1	Reference		—	—	
No	0.831	0.473-1.462		—	—	
**pT status**			0.042			0.012
pT1	0.389	0.114-1.329		0.158	0.015-1.617	
pT2	0.282	0.095-0.841		0.111	0.015-0.834	
pT3	0.759	0.038-1.702		0.840	0.340-2.076	
pT4	1	Reference		1	Reference	
**pN status**			0.006			0.023
pN0	0.322	0.161-0.646		1.00	Reference	
pN1	0.605	0.313-1.168		0.523	0.240-1.141	
pN2	1.00	Reference		0.889	0.263-1.914	
**pTNM stage**			0.003			0.015
Stage I	0.373	0.180-0.773		0.735	0.352-0.916	
Stage II	0.373	0.174-0.801		0.816	0.485-2.572	
Stage III	1.00	Reference		1.00	Reference	
**Smoking history**			0.069			—
Yes	1.852	0.954-3.596		—	—	—
No	1.00	Reference		—	—	
**Drinking history**			0. 112			—
Yes	3.738	1.347-10.370		—	—	
No	1.00	Reference		—	—	
**ABO blood type**			0.006			0.008
A	0.832	0.416-1.666		0.760	0.365-1.582	
B	0.368	0.165-0.823		0.356	0.155-0.819	
O	0.299	0.128-0.699		0.282	0.114-0.694	
AB	1	Reference		1	Reference	
**Tumor size(cm)**			0.019	—	—	0.241
<3.4	0.622	0.342-1.130		0. 673	0.343-1.323	
≥3.4	1	Reference		1	Reference	
**Serum CEA**			0.196			—
Normal	0.701	0.409-1.201		—	—	
Elevated	1	Reference		—	—	
**CA19-9**			0.038			0.956
Normal	0.854	0.340-2.149		0.434	0.226-1.154	
Elevated	1	Reference		1	Reference	
CA125			0.023			0.031
Normal	0. 475	0.161-0.809		0.854	0.199-3.668	
Elevated	1	Reference		1	Reference	

Abbreviations: BMI=body mass index; 95% CI=95% confidence interval; HR=hazard ratio; pTNM stage=pathological tumour-node-metastasis stage. a:Cox proportional hazards model. b:Chinese definition. c: NCOTARGET. 2015;6(34):36884-9.
